# Short-Video Apps as a Health Information Source for Chronic Obstructive Pulmonary Disease: Information Quality Assessment of TikTok Videos

**DOI:** 10.2196/28318

**Published:** 2021-12-20

**Authors:** Shijie Song, Xiang Xue, Yuxiang Chris Zhao, Jinhao Li, Qinghua Zhu, Mingming Zhao

**Affiliations:** 1 Business School Hohai University Nanjing China; 2 School of Information Management Nanjing University Nanjing China; 3 School of Economics and Management Nanjing University of Science and Technology Nanjing China; 4 Sino-French Engineer School Nanjing University of Science and Technology Nanjing China; 5 Department of Pulmonary and Critical Care Medicine Gaochun People’s Hospital Nanjing China

**Keywords:** COPD, information quality, social media, short-video apps, TikTok

## Abstract

**Background:**

Chronic obstructive pulmonary disease (COPD) has become one of the most critical public health problems worldwide. Because many COPD patients are using video-based social media to search for health information, there is an urgent need to assess the information quality of COPD videos on social media. Recently, the short-video app TikTok has demonstrated huge potential in disseminating health information and there are currently many COPD videos available on TikTok; however, the information quality of these videos remains unknown.

**Objective:**

The aim of this study was to investigate the information quality of COPD videos on TikTok.

**Methods:**

In December 2020, we retrieved and screened 300 videos from TikTok and collected a sample of 199 COPD-related videos in Chinese for data extraction. We extracted the basic video information, coded the content, and identified the video sources. Two independent raters assessed the information quality of each video using the DISCERN instrument.

**Results:**

COPD videos on TikTok came mainly from two types of sources: individual users (n=168) and organizational users (n=31). The individual users included health professionals, individual science communicators, and general TikTok users, whereas the organizational users consisted of for-profit organizations, nonprofit organizations, and news agencies. For the 199 videos, the mean scores of the DISCERN items ranged from 3.42 to 4.46, with a total mean score of 3.75. Publication reliability (*P*=.04) and overall quality (*P*=.02) showed significant differences across the six types of sources, whereas the quality of treatment choices showed only a marginally significant difference (*P*=.053) across the different sources.

**Conclusions:**

The overall information quality of COPD videos on TikTok is satisfactory, although the quality varies across different sources and according to specific quality dimensions. Patients should be selective and cautious when watching COPD videos on TikTok.

## Introduction

Chronic obstructive pulmonary disease (COPD) has become one of the most critical public health problems, which has resulted in huge health care expenditures [1]. In 2015, COPD caused approximately 3.17 million deaths worldwide, which accounted for 5% of global deaths for that year [2]. COPD is notably more severe in low- and middle-income countries than in high-income countries. From 2014 to 2015, approximately 5.9% of adults in the United States were reported to be living with COPD [3], whereas the estimated prevalence was 13.6% in China during the same period [4].

COPD is a preventable and treatable disease, and there are many opportunities to reduce the risk of COPD before and after diagnosis [5,6]. For example, general health consumers could reduce the risk of contracting COPD by quitting smoking and avoiding secondhand smoke [7]. Even after a COPD diagnosis, maintaining a healthy lifestyle can help patients prevent exacerbations and improve well-being [8]. However, limited access to information about COPD has become a significant problem for patients and their caregivers [9]. People living with COPD often report limited knowledge on several points such as the causes of COPD and its consequences [10]. Patients have also often received inadequate guidance about how to recognize the disease, and avoid and manage exacerbations [10,11]. Therefore, effective health communications that inform patients on recommended actions are important for better disease prevention and management, and information communication technologies can play a substantial role in such communication and intervention [12].

Emerging technologies provide great health communication opportunities that can inform and empower COPD patients in regard to disease management [13]. For example, mobile technologies have been extensively used to help COPD patients achieve an early diagnosis [14], make medical appointments [15], promote physical activity [16], consistently self-monitor [17], enhance self-management [18], and reduce COPD exacerbation [12]. Recently, visually rich social media (eg, YouTube, Pinterest) have become popular among COPD patients [19,20]. In general, rich social media have several advantages in health communication. The information can be illustrated, which makes it easier to process and remember than information in the form of plain text [21]. Imagistic health information can elicit affective reactions and motivate consumers’ health actions [22]. Some prior studies suggest that COPD patients exposed to visually rich social media are more willing to engage with COPD-related messages [19,23].

Despite the promising potential of any emerging technology, patients’ actual use of a technology can be fraught with problems. Some evidence suggests that information quality is one of the most significant concerns when COPD patients seek health information online [24]. Evaluating the quality of online health information sources is not an easy task for most laypeople [25,26], especially for many COPD patients who have low health literacy [27]. According to a survey of COPD patients, approximately half of the respondents reported difficulty distinguishing between high- and low-quality health resources on the internet [27]. Therefore, health care providers should assess the information quality of online COPD information and advise their patients about it.

To the best of our knowledge, the quality of online COPD information in video-based social media has yet to be sufficiently investigated. Stellefson et al [20] reviewed 223 videos employing the instruments of HONcode (Health on the Net) principles; they found that the majority of YouTube COPD videos (69.1%) were of high quality and were mainly created by authoritative sources (eg, health agencies, organizations, news agents, and professionals). Recently, the short-video app TikTok has attracted significant research attention in regard to its health communication. For example, during the COVID-19 pandemic, TikTok’s coronavirus-related videos were viewed 93.1 billion times as of July 2020 [28]. The originality, interactivity, and sociable nature of TikTok have given the younger generation a better user experience and sense of engagement while seeking health information [29]. TikTok affords rich information modalities (eg, text, image, audio, and video), and contains ample technology features such as commenting, chatting, following, liking, and live-streaming [30]. These features make the app easier for the general public to use as a source of health information, and penetration and usage of TikTok are also on the rise among some older age groups [31].

We observed that there are many COPD-related videos on TikTok. However, the quality of the information they offer remains unclear. Therefore, to fill this gap, the aim of this study was to evaluate the information quality of COPD videos on TikTok.

## Methods

### Search Strategy and Data Extraction

We employed three Chinese words, “慢性阻塞性肺疾病” (chronic obstructive pulmonary disease), “慢性阻塞性肺病” (chronic lung disease related to obstructed airflow), and “慢阻肺” (COPD), to retrieve the relevant COPD videos on TikTok. In its search function, TikTok provides three ways to sort items: “overall ranking,” “most recent,” and “most likes.” Overall ranking is the default mode of sorting recommended by TikTok, which also comprises the other two modes. Given that most users employ the default, we used the overall ranking mode to retrieve the top 100 videos posted from December 6 to December 10, 2020, under each of the three keywords, which resulted in a total of 300 videos. We chose the threshold number of 100 for two reasons. First, TikTok’s search function encompasses the consideration of topic relevance; the pertinent COPD videos mostly appear at the top of the result list, and it is hard to observe any relevant videos when the results go beyond 100. Second, most general health consumers apply the “least effort” principle in their online information-seeking activities; thus, they usually view the top search results instead of going very far [32].

**Figure 1:**
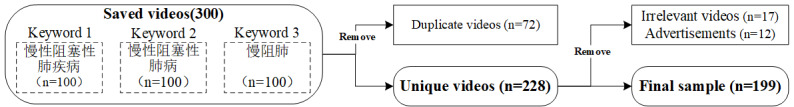
Search strategy and video screening procedure.

To choose the most relevant videos, we removed videos that were (1) duplicates (n=72), (2) not directly related to COPD topics (n=17), and (3) advertisements (n=12). Finally, a total of 199 videos remained for data analysis (see Figure 1).

We used Microsoft Excel to extract and code the basic information from each video. This included a description of the video; the URL; the upload date; the duration (in seconds); the user ID of the uploader; and the numbers of views, likes, and comments.

### Instruments

We employed DISCERN as the instrument for assessing the quality of the information in each video. DISCERN is a brief questionnaire designed to help health consumers and researchers assess the quality of health information. The questionnaire contains three parts, devoted to the reliability of a publication (8 items), the quality of information on treatment choices (7 items), and an overall rating of the publication (1 item) [33]. Each of the 16 questions is rated on a scale of 1 (lowest score) to 5 (highest score). We chose DISCERN for several reasons: (1) it is one of the most widely used instruments for studying the quality of health information [34], (2) it has proven to be effective in Chinese contexts [35], and (3) it has proven to be useful for assessing information quality on other video-based platforms (eg, YouTube) [36]. The full instrument is presented in Multimedia Appendix 1.

Moreover, we adopted six questions from Goobie et al [37] to evaluate the video content. These six questions ask to what degree a video addresses the definition of a disease, its signs and symptoms, risk factors, evaluation, management, and outcomes. Each aspect was scored on a three-item scale: not addressed (0 points), partially addressed (1 point), and sufficiently addressed (2 points).

### Coding Procedure

Two raters (MZ and YZ) independently evaluated the information quality of each video, employing the DISCERN instrument. Both raters are certified physicians who worked in a local hospital in the division of respiratory disease. The coding procedure contained three stages.

In the first stage, we recorded the basic information of the video publishers (eg, account name, self-description, identity verification status) and the basic information of the videos (eg, publication date, video length, number of likes, number of comments, number of shares). Regarding the video publishers, we categorized the video sources into two main types (ie, individual users and organizational users) by their account names and identity verification status. Further, we identified several subcategories within each source type by their account names, self-description, and video publication records. For example, if a video publisher described themselves as a “scientific writer,” we would code the source as an “individual science communicator.”

In the second stage, we assessed the video content using the six categories from Goobie et al [37]: the definition of a disease, its signs and symptoms, risk factors, evaluation, management, and outcomes. When we had independently scored the first 30 videos, we found that we were able to reach a consensus on whether a video contains a certain category of content. One rater (MZ) then finished rating the rest of the videos and the other rater (YZ) validated the codes, followed by discussion and resolution of any inconsistencies between the two raters.

In the third stage, we evaluated the information quality by applying the DISCERN instrument. Before starting to score the videos, the two raters first reviewed the official DISCERN scoring instructions, discussing how the tool could be operationalized for evaluating video-based content and making necessary adjustments. Using the DISCERN questions, the two raters scored all videos independently. The overall rating agreement (Cohen κ) was 0.793 (*P*<.001), which indicated that the rating process had satisfactory interrater reliability. Any between-group analyses regarding DISCERN scores were performed using the Kruskal-Wallis H test in SPSS 22.

## Results

### Video Characteristics

The COPD videos on TikTok came mainly from two types of sources: individual users and organizational users. Most of the COPD TikTok videos in our sample were contributed by individual users (168/199, 84.4%), whereas a relatively small share was contributed by organizational users (31/199, 15.6%). For each type, we identified three subcategories (see Table 1). Among individual users, health professionals created the most videos, followed by individual science communicators and general TikTok users. Among organizational users, for-profit organizations published the highest number of videos, followed by nonprofit organizations and news agencies.

In the sample, the durations of the videos varied from 10 to 4116 seconds. The videos published by nonprofit organizations were significantly longer than videos from other sources, whereas the videos published by news agencies had the shortest average duration. Videos published by individual science communicators had the second-longest average duration. The average duration for the other sources was under 1 minute (Table 2).

The most recent video was published 22 days prior to data collection, whereas the oldest had been on TikTok for more than 1 year. The 199 videos received a total of 1,696,725 likes and 175,703 comments prior to data collection. The number of likes varied from 0 to 662,000 for each video, and the number of comments ranged from 0 to 18,000. The videos published by health professionals received the most likes and comments, whereas the videos uploaded by individual science communicators received the least likes and comments. Since their publication, the videos in the overall sample had been shared a total of 167,473 times. The videos uploaded by health professionals were shared the most frequently, whereas the videos created by individual science communicators were shared the least frequently, as shown in Table 2.

**Table 1:** Characteristics of the sources of chronic obstructive pulmonary disease–related TikTok videos (N=199).

Source		Description	Videos, n (%)
**Individual users**			
	General users	Common TikTok users	30 (15.1)
	Individual science communicators	Individuals who participate in general scientific communications, which may include but are not limited to health care domains	39 (19.6)
	Health professionals	Individuals who describe themselves as health professionals	99 (49.7)
**Organizational users**			
	For-profit organizations	Private sector organizations	18 (9.0)
	Nonprofit organization	Organizations or hospitals operating in the public sector	8 (4.0)
	News agencies	Organizations providing news services	5 (2.5)

**Table 2:** Characteristics of chronic obstructive pulmonary disease–related TikTok videos, by source

Source of videos		Days on TikTok, median (IQR)	Video duration (seconds), median (IQR)	Number of likes, median (IQR)	Number of comments, median (IQR)	Number of shares, median (IQR)
**Organizational users**						
	For-profit organizations	180 (39-297)	59 (37-129)	364 (115-4915)	17 (7-32)	19 (7-133)
	Nonprofit organizations	148 (44-225)	158 (74-206)	24 (14-40)	1 (0-2)	1 (0-8)
	News agencies	145 (22-147)	34 (18-51)	12 (1-18)	0 (0-0)	3 (1-3)
**Individual users**						
	Health professionals	407 (144-490)	57 (49-59)	1031 (170-3203)	54 (8-161)	58 (12-334)
	Individual science communicators	303 (147-491)	85 (45-116)	3 (1-11)	0 (0-1)	0 (0-5)
	General users	237 (133-394)	40 (15-59)	19 (9-49)	2 (1-5)	1 (0-7)
**Overall**		276 (119-478)	57 (43-88)	125 (12-1597)	7 (1-91)	12 (1-118)

### Information Quality

**Figure 2:**
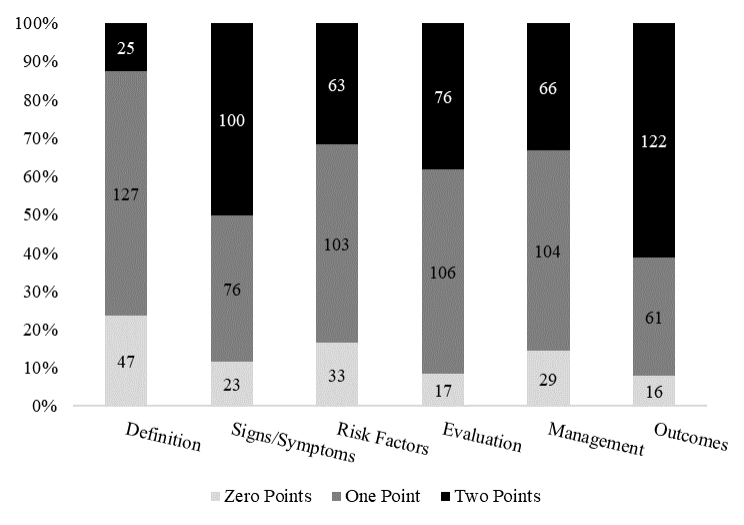
Percentage of videos addressing each chronic obstructive pulmonary disease topic.

The videos covered the six predefined content areas to different degrees, as shown in Figure 2. The results suggested that more than half of the videos (122/199, 61.3%) sufficiently addressed COPD outcomes, whereas only 16 (8.0%) made no mention of outcomes. The second most frequently introduced area concerned the signs or symptoms of COPD, with almost half of the videos (100/199, 50.3%) sufficiently addressing them and 76 (38.2%) only giving partial mentions. Moreover, approximately half of the videos only partially mentioned the topics of COPD evaluation, management, and risk factors. The least frequently mentioned topic was the definition of COPD; only 25 videos (12.6%) sufficiently addressed this and 47 videos (23.6%) did not mention it at all.

We calculated the mean scores for each DISCERN item for the total sample. The scores ranged from 3.42 to 4.46 (mean 3.75). The scores of the eight items measuring publication reliability (items 1-8) varied from 3.42 to 4.46 (mean 3.90). For the seven items assessing the quality of information on treatment choices (items 9-15), the scores ranged from 3.45 to 3.69 (mean 3.56). The remaining item (item 16) measuring overall information quality achieved a mean of 3.85 out of 5 points. We categorized the DISCERN items into three sections according to the original instrument indicated: reliability of the publication, quality of information on treatment choices, and overall rating of the publication (see Table 3).

Videos published by nonprofit organizations had the highest reliability, whereas the videos contributed by general users had the lowest reliability. The differences in publication reliability across the six types of video sources were statistically significant. Regarding the quality of information on treatment choices, the nonprofit organizations provided the highest-quality videos, whereas the general users provided the lowest-quality videos; however, the differences among the various information sources were only marginally significant. With regard to the last item concerning overall rating, the highest-quality videos were created by news agencies and the lowest-quality videos were generated by for-profit organizations. The overall rating of information quality showed significant differences according to different sources. Overall, the total scores of the entire DISCERN questionnaire across the different sources exhibited significant differences (Table 3).

**Table 3:** DISCERN scores of chronic obstructive pulmonary disease–related TikTok videos by source (N=199).

Category		For-profit organizations (n=18)	Nonprofit organizations (n=8)	News agencies (n=5)	Health professionals (n=99)	Science communicators (n=39)	General users (n=30)	*P* value^a^	
**Publication reliability**									.04
	Median	30.5	34.5	34	32	33	30		
	Mean (SD)	29.8 (5.9)	34.5 (1.31)	34.1 (1.4)	31.1 (3.7)	31.1 (4.4)	29.7 (5.8)		
**Treatment choices**									.053
	Median	24.3	28	26	25.5	26	23		
	Mean (SD)	22.8 (5.7)	28.1 (2.93)	27.3 (3.9)	25.3 (4.2)	25 (4.5)	23.1 (6.1)		
**Overall quality**									.02
	Median	4	4	4	4	4	4		
	Mean (SD)	3.4 (0.9)	4.2 (0.65)	4.4 (0.5)	3.8 (0.6)	3.9 (0.7)	3.6 (0.9)		
**Total score**									.01
	Median	60	67	66	61	62	57		
	Mean (SD)	56 (11.8)	66.8 (3.8)	65.8 (4.5)	60.4 (7.3)	60 (8.9)	56.5 (11.1)		

^a^*P* values were calculated with the Kruskal-Wallis H test.

## Discussion

### TikTok as a Health Information Source

Recently, video-based social media platforms and apps have been gaining popularity among patients with chronic conditions [38]. For example, YouTube has become a prominent platform for generating and spreading health-related videos, covering topics related to chronic disease management, including disease prevention, diagnosis, and treatment [39]. Although some recent evidence indicates that TikTok has had vast communication potential during the COVID-19 pandemic [40], the role of TikTok in disseminating chronic disease information remains unclear.

Our results suggest that TikTok could be a promising channel for disseminating COPD information. The 199 videos surveyed in our study have received approximately 1.7 million likes and 176,000 comments since they were published. Given that most of the videos were published within 1 year, the numbers of likes and comments are relatively high. Therefore, we suggest that health care professionals and institutions leverage short-video apps (eg, TikTok) to improve patient education and health communication.

### Information Quality

To the best of our knowledge, the quality of online information about COPD is understudied. Specifically in regard to short-video apps, the information quality of COPD videos remains unclear. As one of the earliest studies to tackle this problem, this study yielded results suggesting that the general information quality of COPD videos on TikTok is relatively satisfactory. Although there is a concern that TikTok may differentiate itself from other social media by targeting quirky videos rather than serious professional content [30], our results indicate that the quality of health information found on TikTok, particularly that related to COPD, is acceptable. These results are consistent with a recent survey of coronavirus-related videos on TikTok, which found that the information provided in these videos was generally credible, containing merely 4.3% misinformation [41].

Our findings show that the COPD videos on TikTok more or less touched on all of the preidentified COPD-related content. More than half of the videos sufficiently described the outcomes and the signs and symptoms, and partially addressed the topics of evaluation, risk factors, and management. However, few videos offered definitions of COPD. A possible explanation for this is that TikTok videos are created for a target audience of laypeople. Therefore, introducing aspects of disease management is more important than discussing academic definitions of COPD.

### Sources of COPD-Related Videos on TikTok

This study reveals that both individual users and organizational users are engaged in creating COPD videos on TikTok. We identified three specific subcategories for each of these two general categories of sources (ie, individual users and organizational users). Individual users included health professionals, general TikTok users, and individual science communicators. We found that many health professionals were active in communicating COPD knowledge; they contributed almost half of the videos in our sample. Organizational communicators of health information included nonprofit organizations, for-profit organizations, and news agencies. Recently, some researchers have suggested that video-based social media such as TikTok are playing increasingly important roles in providing general health information sought or encountered by consumers [40,41]; therefore, health experts and traditional institutions should harness and leverage social media platforms and apps to better disseminate health information and medical knowledge to the public [41,42]. In general, the findings of this study indicate that health professionals and reputable organizations are actively engaged in communicating health information on TikTok.

In addition, the results suggest that the information quality of COPD videos on TikTok varies according to the source. The videos published by nonprofit organizations had the highest average score for the reliability of publications, while the videos created by general TikTok users had the lowest average score; the differences were statistically significant. The nonprofit organizations and general TikTok users earned the highest and lowest average scores for the quality of information on treatment choices, respectively, although the differences were only marginally significant. For the overall rating of information quality, the news agencies contributed content of significantly higher quality than that of other sources. All of these results are consistent with prior studies in YouTube settings, where organizational users were found to create videos of significantly higher quality than those of individual users [20,37].

Communication performance varied among the sources. In our sample, the videos published by health professionals received the most likes and comments, and were most frequently shared by users. The videos created by individual science communicators received the least likes and comments and were least likely to be shared by users. Interestingly, despite the significant differences in communication performance, we found that the two sources had comparable levels of objective information quality, as assessed by the DISCERN instrument. Prior information credibility studies suggest that credibility depends on the perception of the information recipient, which may not necessarily reflect the objective quality of the information [25,43], and users’ credibility perception predicts whether or not they will adopt and share health information on social media [44]. The discrepancy between information quality and communication performance may partially confirm such findings. We suspect that health professionals convey a higher level of expertise and thus generate greater credibility, whereas general science communicators convey low expertise in medicine when they upload videos with wide topic coverage extending beyond the medical domain. We call for future research to investigate the discrepancies between the actual quality of health information and users’ credibility perception.

### Limitations and Future Research

This study has some limitations. First, we employed only the DISCERN instrument, which was chosen because it has worked well in prior studies that assessed the quality of information in health-related videos. However, there are other instruments available, such as the JAMA (Journal of the American Medical Association) benchmarks and the HONcode, and future research could expand these investigations by using different instruments. Second, this study only assessed the information quality of Chinese COPD videos. Although the locality did not impact the overall results, the conclusions may not necessarily be generalizable to COPD videos in other languages; therefore, we call for more cross-language comparative studies in the future.

### Conclusion

This study investigated the information quality of COPD videos on the world’s largest short-video app, TikTok, employing the DISCERN instrument. The results show that both organizational and individual users generate COPD-related content. The overall information quality of the COPD-related videos in the sample was satisfactory, although the quality varied across the different video sources and specific quality dimensions. The results suggest that patients should remain cautious and selective when watching COPD videos on TikTok. Videos from identifiable sources (eg, nonprofit organizations) are much more strongly recommended than those from other nonverified sources. Based on the limitations of this study, we have proposed several directions for future research.

## Appendix

Multimedia Appendix 1 The DISCERN questionnaire.

## Abbreviations

COPD: chronic obstructive pulmonary disease
HON: Health on the Net
JAMA: Journal of the American Medical Association
