# Supplementary material for: Short-Video Apps as a Health Information Source for Chronic Obstructive Pulmonary Disease: Information Quality Assessment of TikTok Videos
Source: J Med Internet Res. 2021 Dec 20;23(12):e28318. doi: 10.2196/28318 (PMC8726035; doi:10.2196/28318)
Supplement: Multimedia Appendix 1 [file jmir_v23i12e28318_app1.docx]

The DISCERN questionnaire

| 1 | Are the aims clear? |
| --- | --- |
| 2 | Does it achieve its aims? |
| 3 | Is it relevant? |
| 4 | Is it clear what sources of information were used to compile the publication (other than the author or producer)? |
| 5 | Is it clear when the information used or reported in the publication was produced? |
| 6 | Is it balanced and unbiased? |
| 7 | Does it provide details of additional sources of support and information? |
| 8 | Does it refer to areas of uncertainty? |
| 9 | Does it describe how each treatment works? |
| 10 | Does it describe the benefits of each treatment? |
| 11 | Does it describe the risks of each treatment? |
| 12 | Does it describe what would happen if no treatment is used? |
| 13 | Does it describe how the treatment choices affect overall quality of life? |
| 14 | Is it clear that there may be more than one possible treatment choice? |
| 15 | Does it provide support for shared decision making? |
| 16 | Based on the answers to all of these questions, rate the overall quality of the publication as a source of information about treatment choices |
